# JNK suppression is essential for 17β-Estradiol inhibits prostaglandin E2-Induced uPA and MMP-9 expressions and cell migration in human LoVo colon cancer cells

**DOI:** 10.1186/1423-0127-18-61

**Published:** 2011-08-22

**Authors:** Hsi-Hsien Hsu, Wei-Syun Hu, Yueh-Min Lin, Wei-Wen Kuo, Li-Mien Chen, Wei-Kung Chen, Jin-Ming Hwang, Fuu-Jen Tsai, Chung-Jung Liu, Chih-Yang Huang

**Affiliations:** 1 Division of Colorectal Surgery, Mackay Memorial Hospital, Taipei, Taiwan; 2 Mackay Medicine, Nursing and Management College, Taipei, Taiwan; 3 Division of Cardiology, Taipei Medical University Shuang-Ho Hospital, Taipei, Taiwan; 4 Graduate Institute of Aging Medicine, China Medical University, Taichung, Taiwan; 5 School of medicine, Chung Shan Medical University, Taichung, Taiwan; 6 Department of Medical Technology, Jen-Teh Junior College of Medicine, Nursing and Management, Miaoli, Taiwan; 7 Department of Biological Science and Technology, China Medical University, Taichung, Taiwan; 8 Division of Medical Technology, Department of Internal Medicine, Armed-Force, Taichung General Hospital, Taichung, Taiwan; 9 Emergency Department, China Medical University Hospital, Taichung, Taiwan; 10 School of Applied Chemistry, Chung Shan Medical University, Taichung, Taiwan; 11 Department of Pediatrics, Medical Research and Medical Genetics, China Medical University, Taichung, Taiwan; 12 Division of Gastroenterology, Department of Internal Medicine, Kaohsiung Medical University Hospital, Kaohsiung, Taiwan; 13 Cancer Center, Kaohsiung Medical University Hospital, Kaohsiung, Taiwan; 14 Graduate Institute of Basic Medical Science, China Medical University, Taichung, Taiwan; 15 Department of Health and Nutrition Biotechnology, Asia University, Taichung, Taiwan

## Abstract

**Background:**

Epidemiological studies demonstrate that the incidence and mortality rates of colorectal cancer in women are lower than in men. However, it is unknown if 17β-estradiol treatment is sufficient to inhibit prostaglandin E2 (PGE2)-induced cellular motility in human colon cancer cells.

**Methods:**

We analyzed the protein expression of urokinase plasminogen activator (uPA), tissue plasminogen activator (tPA), matrix metallopeptidases (MMPs), plasminogen activator inhibitor-1 (PAI-1) and tissue inhibitor of metalloproteinases (TIMPs), and the cellular motility in PGE2-stimulated human LoVo cells. 17β-Estradiol and the inhibitors including LY294002 (Akt activation inhibitor), U0126 (ERK1/2 inhibitor), SB203580 (p38 MAPK inhibitor), SP600125 (JNK1/2 inhibitor), QNZ (NFκB inhibitor) and ICI 182 780 were further used to explore the inhibitory effects of 17β-estradiol on PGE2-induced LoVo cell motility. Student's t-test was used to analyze the difference between the two groups.

**Results:**

Upregulation of urokinase plasminogen activator (uPA), tissue plasminogen activator (tPA) and matrix metallopeptidases (MMPs) is reported to associate with the development of cancer cell mobility, metastasis, and subsequent malignant tumor. After administration of inhibitors including LY294002, U0126, SB203580, SP600125 or QNZ, we found that PGE2 treatment up-regulated uPA and MMP-9 expression via JNK1/2 signaling pathway, thus promoting cellular motility in human LoVo cancer cells. However, PGE2 treatment showed no effects on regulating expression of tPA, MMP-2, plasminogen activator inhibitor-1 (PAI-1), tissue inhibitor of metalloproteinase-1, -2, -3 and -4 (TIMP-1, -2, -3 and -4). We further observed that 17β-estradiol treatment inhibited PGE2-induced uPA, MMP-9 and cellular motility by suppressing activation of JNK1/2 in human LoVo cancer cells.

**Conclusions:**

Collectively, these results suggest that 17β-estradiol treatment significantly inhibits PGE2-induced motility of human LoVo colon cancer cells.

## Background

Colorectal carcinoma (CRC) is one of the most prevalent cancers world-wide [1], and is the secondary leading cause of cancer-related mortality in the developed countries [2]. Colon cancer accounts for more than 130,000 new cases per year [3] and causes more than 56,000 deaths per year in United States [4] despite the advanced chemotherapeutic treatments.

Degradation of extracellular matrix (ECM) is closely associated the development of malignant tumor. ECM degradation by extracellular proteinases accelerates the progress of tumor cell invasion and metastasis [5]. The proteolytic proteinase systems primarily responsible for ECM degradation in vivo are matrix metalloproteinase (MMPs) and plasminogen activator (PA) systems [5,6]. Matrix metalloproteinases (MMPs) are a family of functionally related zinc-containing enzymes that include interstitial collagenases, gelatinases, stromelysin, matrilysin, metalloelastase, and membrane-type MMPs [7,8]. Upregulation of MMP-2 and MMP-9 has been shown to play a key role in the progression, invasion, metastasis of colorectal cancer in animal models and patients [9]. MMP activity is closely controlled by physiological inhibitors, TIMPs including TIMP-1, -2, -3 and -4 [10]. Another proteolytic plasminogen system with its plasminogen activators (PA), such as urokinase-type plasminogen activators (uPA) and tissue-type plasminogen activators (tPA) is showed to activate MMPs and to be involved in colon cancer progression [11]. Upregulation of uPA and tPA is considered as a marker of several types of malignant cancer including colon cancer [12].

Epidemiological studies demonstrate that the incidence and mortality rates of colorectal cancer in women are lower than in men [13]. Estrogen (E_2_) performs the profound effects on target tissue is mediated by two estrogen receptor (ER) subtypes ERα and ERβ [14]. ERα and ERβ have been identified in colon tissue in both sexes [15]. In observational studies, estrogen exerts a protective role against the development of fatal colon cancer with a substantially decreased risk in women receiving hormone replacement therapy (HRT) [16-18], and a reduced mortality from this disease [19]. However, the precise mechanism behind protective effects of 17β-estradiol against PGE2-induced progression in colon cancer remains unclear. In the present study, we examined the effects of 17β-estradiol on PGE2-induced cellular motility in human LoVo colon cancer cells, and further identified the precise molecular and cellular mechanisms behind this protective property. The results demonstrated that 17β-estradiol treatment inhibits PGE2-induced cellular motility and expression of uPA and MMP-9 by suppressing the activation of JNK1/2 in LoVo cells. The present study suggests that 17β-estradiol presents the properties of anti-cancer by inhibiting PGE2-induced migration in human LoVo cancer cells.

## Materials and Methods

### Cells, Antibodies, Reagents and Enzymes

Human colon cancer cell lines, LoVo, were obtained from the American Tissue Culture Collection (ATCC) (Rockville, MD, USA). LoVo cells were established from the metastatic nodule resected from a 56-year-old colon adenocarcinoma patient. 17β-estradiol (E_2_) and hydroxyurea were purchased from Sigma (Sigma Chemical Co., St. Louis, Missouri, USA). Prostaglandins E2 (PGE2) was purchased from CALBIOCHEM (Darmstadt, Germany). The LY294002 (PI3K inhibitor), U0126 (MEK1/2 inhibitor), SB203680 (p38 MAPK inhibitor), SP600125 (JNK inhibitor), and ER antagonist ICI 182,780 (ICI) were purchased from TOCRIS (Ellisville, Missouri, USA). 6-Amino-4-(4-phenoxyphenylethylamino) quinazoline (QNZ), NFκB activation inhibitor was purchased from Peptides International (Louisville, Kentucky, USA). We utilized the following antibodies against JNK1/2, phospho-JNK1/2, uPA, tPA, PAI-1, MMP-2, MMP-9, TIMP-1, TIMP-2, TIMP-3 and TIMP-4 (Santa Cruz Biotechnology, Inc. Santa Cruz, California, USA); α-tubulin (Lab Vision Corporation, Fremont, California, USA) as loading control. Goat anti-mouse IgG antibody conjugated to horseradish peroxidase and goat anti-rabbit IgG antibody conjugated to horseradish peroxidase and rabbit anti-goat IgG horseradish peroxidase conjugate were purchased from Santa Cruz Biotechnology, Inc. in California, USA.

### Cell Culture

LoVo colon cancer cell line from the American Type Culture Collection (ATCC) (Rockville, MD) were cultured on 100-mm or 60-mm culture dishes in Dulbecco's modified Eagle's medium (DMEM) supplemented with 100 μg/ml penicillin, 100 μg/ml streptomycin, 2 mM glutamine, 1 mM HEPS buffer, and 10% Clontech fetal bovine serum in humidified air (5% CO_2_) at 37°C

### Immunoblotting

To isolate total proteins, cultured LoVo cells were washed with cold PBS and resuspended in lysis buffer (50 mM Tris, pH 7.5, 0.5 M NaCl, 1.0 mM EDTA, pH 7.5, 10% glycerol, 1 mM BME, 1% IGEPAL-630 and a proteinase inhibitor cocktail (Roche Molecular Biochemicals)). After incubation for 30 min on ice, the supernatant was collected by centrifugation at 12000 g for 15 min at 4°C, and the protein concentration was determined by the Bradford method. Sample containing equal proteins (60 μg) were loaded and analyzed by Western blot analysis. Briefly, proteins were separated by 12% SDS-PAGE and transferred onto PVDF membrane (Millipore, Belford, Massachusetts, USA). Membrane were blocked with blocking buffer (5% non-fat dry milk, 20 mM Tris-HCl, pH 7.6, 150 mM NaCl, and 0.1% Tween 20) for at least 1 h at room temperature. Membranes were incubated with primary antibodies in the above solution on an orbit shaker at 4°C overnight. Following primary antibody incubation, membranes were incubated with horseradish peroxidase-linked secondary antibodies (anti-rabbit, anti-mouse, or anti-goat IgG).

### Migration Assay

Migration assay was performed using the 48-well Boyden chamber (Neuro Probe) plate with the 8-μm pore size polycarbonate membrane filters [20]. The lower compartment was filled with DMEM containing 20% FCS. LoVo cells were placed in the upper part of the Boyden chamber containing serum-free medium and incubated for 48 h. After incubation, the cells on membrane filter were fixed with methanol and stained with 0.05% Giemsa for 1 h. The cells on upper surface of the filter were removed with a cotton swab. The filters were then rinsed in double distilled water until additional stain was leached. The cells then were air-dried for 20 min. The migratory phenotypes were determined by counting the cells that migrated to the lower side of the filter with microscopy at 200× and 400× magnification, respectively. The fourth fields were counted for each filter, and each sample was assayed in triplicate.

### Statistical Analysis

Each experiment was duplicated at least three times. Results were presented as the mean ± SE, and statistical comparisons were made using the Student's *t *test. Significance was defined at the p < 0.05 or 0.01 levels.

## Results

### The Effects of Prostaglandin E2 on Expression of uPA, tPA, MMP-2 and MMP-9 in Human LoVo Colon Cancer Cells

**Figure 1:**
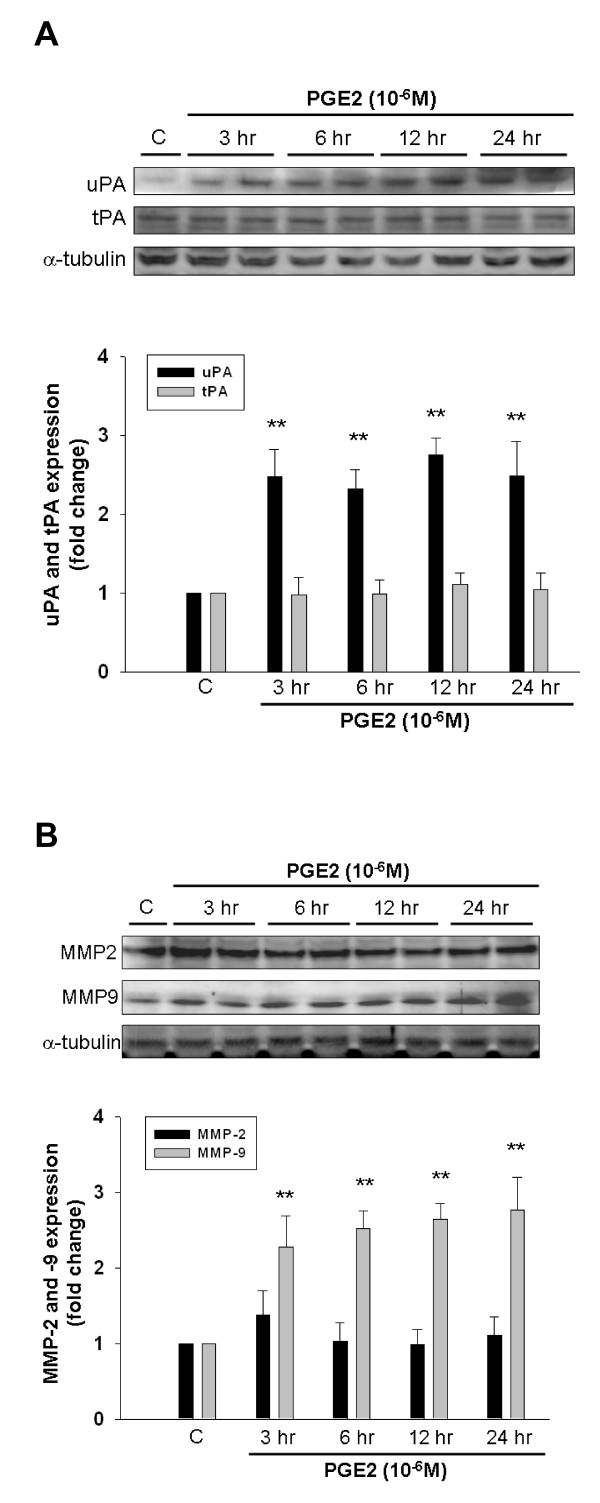
**PGE2 induces expression of uPA and MMP-9 in human LoVo colon cancer cells**. LoVo cells cultured in DMEM were treated with PGE2 (10^-6^M) for 3 h, 6 h, 12 h and 24 h, and subsequently observed protein level of uPA, tPA **(A)**, MMP-2 and MMP-9 **(B) **in LoVo cells by immunoblotting assay. The responses to different time periods of PGE2 treatment were measured by the immunoblotting assay. **, *p *< 0.01 *versus *control (mean ± SE, n = 3).

We detected the expression of cellular migration-regulating factors such as urokinase-type plasminogen activators (uPA), tissue-type plasminogen activators (tPA), matrix metalloproteinases-2 and -9 (MMP-2 and -9) in LoVo cells. Activation of proteolytic plasminogen system with t-PA and u-PA is shown to be involved in upregulation of MMPs [6]. In the present study, we observed that the significant increase in expression levels of uPA (Figure 1A) and MMP-9 (Figure 1B) was induced following PGE2 (10^-6^M) treatment within 3 h, and was maintained up for 24 h. The quantitative results showed that uPA was significantly increased by approximately 2.47-fold within 3 h, 2.32-fold within 6 h, 2.75-fold within 12 h, and 2.48-fold within 24 h. MMP-9 was significantly increased by approximately 2.27-fold within 3 h, 2.52-fold within 6 h, 2.65-fold within 12 h, and 2.79-fold within 24 h. However, PGE2 treatment showed no effects on protein expression of tPA and MMP-2.

### The Effects of Prostaglandin E2 on the Expression of PAI-1 and TIMPs in Human LoVo Colon Cancer Cells

**Figure 2:**
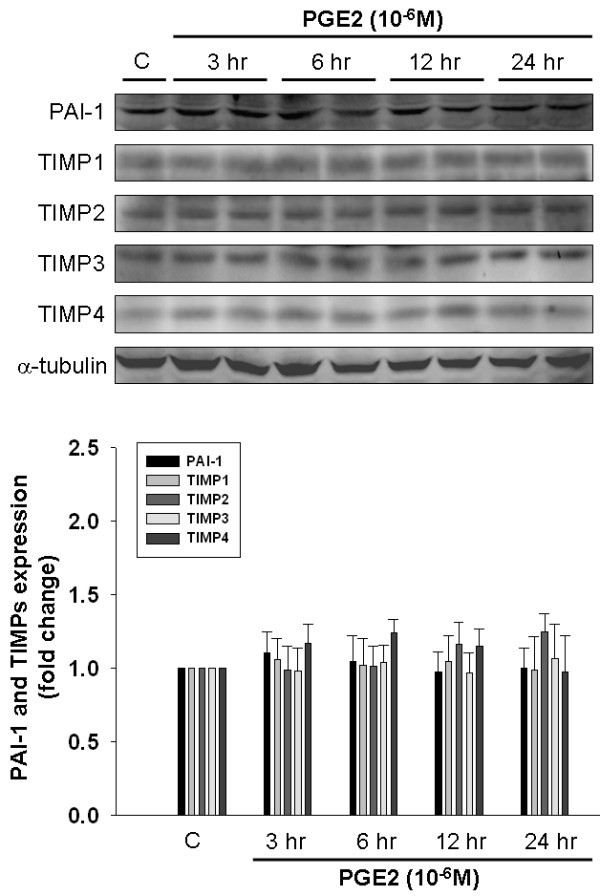
**The effect of PGE2 on the expression of PAI-1 and TIMPs in human LoVo colon cancer cells**. LoVo cells cultured in DMEM were treated with PGE2 (10^-6^M) for 3 h, 6 h,12 h and 24 h, and then were harvested and lysed. Total protein of cell extracts was separated by 12% SDS-PAGE, transferred to PVDF membranes, and immunoblotted with antibodies against proteins as indicated. Equal loading was assessed with an anti-α-tubulin antibody.

uPA and tPA is closely controlled by PAI-1. In addition, activation of MMP-2 and MMP-9 was inhibited by TIMPs, therefore we further examined whether the expression of PAI-1 and TIMPs including TIMP-1, -2, -3, and -4 was reduced by PGE2 treatment. As shown in Figure 2, LoVo cells were treated with PGE2 (10^-6^M) for various periods (3 h, 6 h, 12 h and 24 h), and subsequently subjected to immunoblotting assay. We observed that PGE2 shows no influence on the expression of PAI-1, TIMP-1, -2, -3, and -4 in human LoVo cells.

### JNK1/2 Mediates PGE2-Upregulated uPA and MMP-9 in Human LoVo Colon Cancer Cells

**Figure 3:**
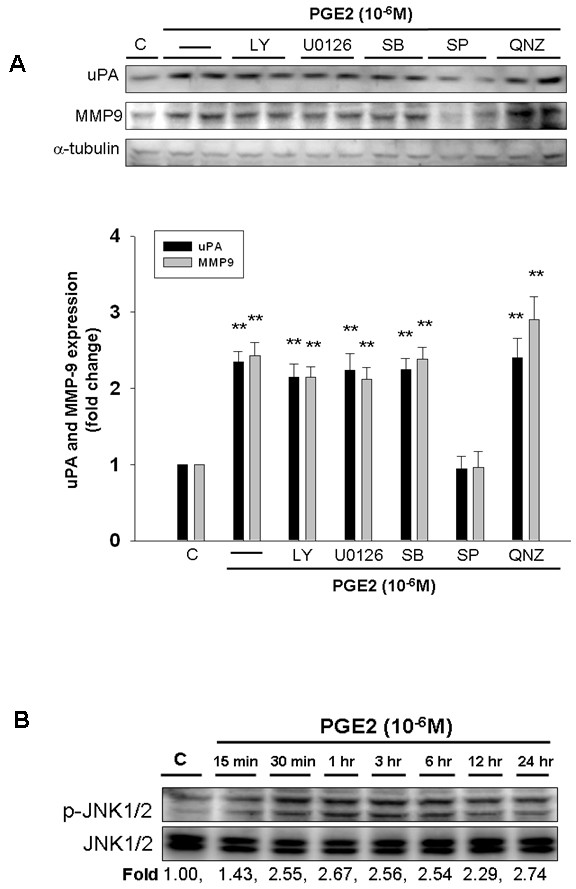
**PGE2 upregulates uPA and MMP-9 via JNK1/2 signaling pathway in human LoVo colon cancer cells**. **(A) **LoVo cells were pretreated with vehicle, LY294002 (Akt activation inhibitor), U0126 (ERK1/2 activation inhibitor, 1 μM), SB203580 (p38 MAPK inhibitor, 1 μM), SP600125 (JNK1/2 inhibitor, 1 μM) or QNZ (NFκB inhibitor, 1 μM) for 1 h and followed by PGE2 (10^-6^M) administration for 24 h, and then were harvested for immunoblotting assays. **(B) **LoVo cells cultured in DMEM were treated with PGE2 (10^-6^M) for various periods (15 min, 30 min, 1 h, 3 h, 6 h, 12 h and 24 h), and subsequently measured the phosphorylation/activation of proteins by immunoblotting assay. The fold ratio of p-JNK1/2 and JNK1/2 was measured. Total protein of cell extracts was separated by 12% SDS-PAGE, transferred to PVDF membranes, and immunoblotted with antibodies against uPA, MMP-9 **(A)**, phospho-JNK1/2 and JNK1/2 **(B) **proteins. Equal loading was assessed with an anti-α-tubulin antibody. **, *p *< 0.01 *versus *control (mean ± SE, n = 3).

To further identify which signal transduction pathway(s) was involved in the mechanism behind PGE2-upregulated expression of uPA and MMP-9 in human colon cancer cells, we applied the following inhibitors such as LY294002 (Akt activation inhibitor), U0126 (ERK1/2 activation inhibitor), SB203580 (p38 MAPK inhibitor), SP600125 (JNK1/2 inhibitor), and QNZ (NFκB activation inhibitor) to respectively block these pathways, followed by the administration of PGE2. LoVo cells were preincubated with LY294002 (1 μM), U0126 (1 μM), SB203580 (1 μM), SP600125 (1 μM) or QNZ (1 μM) for 1 h and followed by the administration of PGE2 (1 μM) for 24 h, and subsequently were subjected to immunoblotting assay to assess the effect of these inhibitors on PGE2-induced expression of uPA and MMP-9. We observed that PGE2-induced expression of uPA and MMP-9 was significantly inhibited by JNK1/2 inhibitor, SP600125, in LoVo cells. The results suggested that PGE2 upregulates expression of uPA and MMP-9 via JNK1/2 signaling pathway in human LoVo colon cancer cells (Figure 3A).

To further explore the effects of PGE2 on activation of JNK1/2 in human LoVo colon cancer cells, we treated LoVo cells with PGE2 (10^-6^M) for various time periods (15 min, 30 min, 1 h, 3 h, 6 h, 12 h and 24 h), and subsequently measured the phosphorylation/activation of proteins by immunoblotting assay. Phosphorylation of JNK1/2 was significantly induced within 15 min in response to PGE2 stimulation, and was maintained up for 24 h (Figure 3B). The findings suggested that administration of PGE2 may induce the motility of human colon cancer by inducing the activation of JNK1/2.

### 17β-Estradiol Inhibits PGE2-Induced Expression of uPA and MMP-9 by Suppressing Activation of JNK1/2

**Figure 4:**
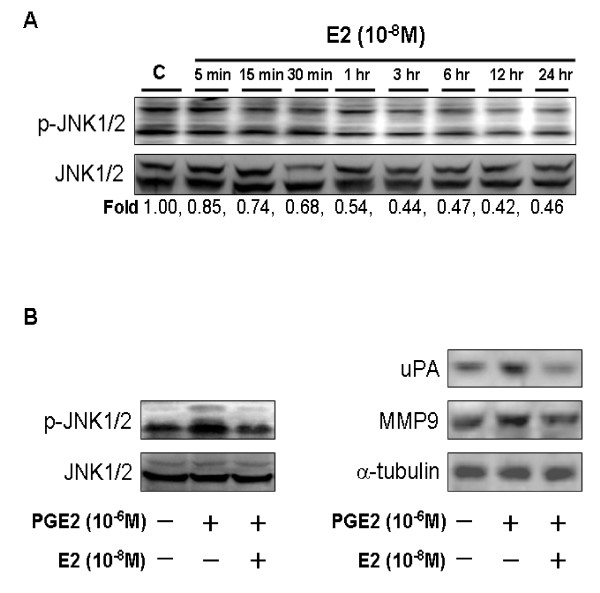
**17β-Estradiol down-regulates PGE2-induced uPA and MMP-9 expression by suppressing activation of JNK1/2 in human LoVo cells**. **(A) **LoVo cells cultured in DMEM were treated with 17β-estradiol (10^-8^M) for various periods (5 min, 15 min, 30 min, 1 h, 3 h, 6 h, 12 h and 24 h), and subsequently measured the phosphorylation/activation of proteins by immunoblotting assay. The fold ratio of p-JNK1/2 and JNK1/2 was measured. **(B) **LoVo cells were pretreated with 17β-estradiol (10^-8^M) for 30 min, followed by PGE2 (10^-6^M) treatment for 30 min or 24 h, and then were subjected to immunoblotting assay for protein detection of phospho-JNK1/2 (PGE2 stimulation within 30 min); uPA and MMP-9 (PGE2 stimulation within 24 h).

In the present study, we treated LoVo cells with 17β-estradiol (10^-8^M) for various time periods (5 min, 15 min, 30 min, 1 h, 3 h, 6 h, 12 h and 24 h), and subsequently measured the phosphorylation/activation of proteins by immunoblotting assay. The results show that phosphorylation of JNK1/2 was significantly reduced within 5 min in response to 17β-estradiol stimulation, and was maintained up for 24 h (Figure 4A). We further examined whether 17β-estradiol inhibits PGE2-induced expression of uPA and MMP-9, and identified the related precise/molecular mechanism in LoVo cells. LoVo cells were pretreated with 17β-estradiol (10^-8^M) for 30 min, followed by PGE2 (10^-6^M) treatment for 30 min or 24 h, and then were subjected to immunoblotting assay for protein detection of phospho-JNK1/2, uPA and MMP-9, respectively. We observed that 17β-estradiol treatment significantly inhibits PGE-induced activation of JNK1/2 within 30 min, and suppressed PGE2-induced expression of uPA and MMP-9 within 24 h in human LoVo colon cancer cells (Figure 4B).

### 17β-Estradiol Inhibits PGE2-Induced Cell Migration in Human LoVo Colon Cancer Cells

**Figure 5:**
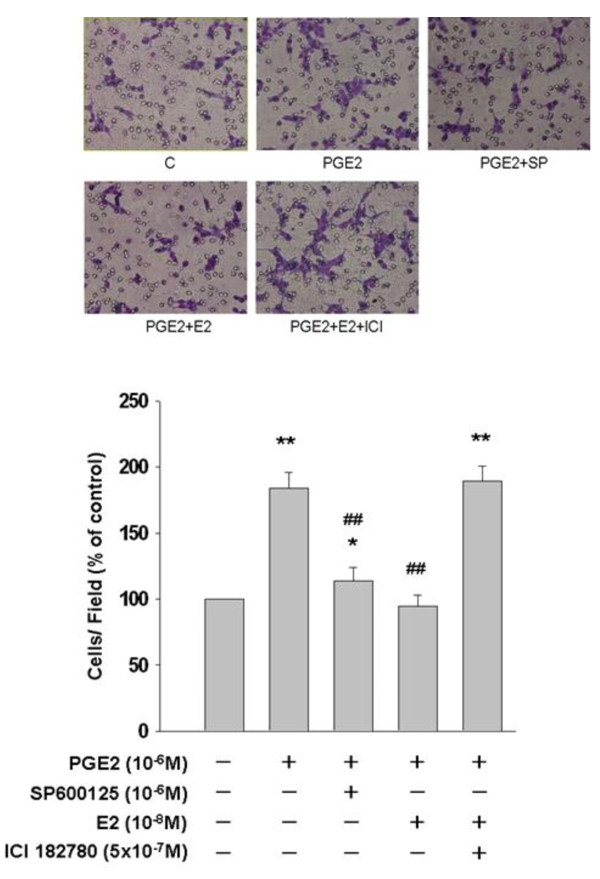
**17β-estradiol inhibits PGE2-promoted cellular motility in human LoVo cells**. LoVo cells were pretreated with vehicle, SP600125 (JNK1/2 inhibitor, 1 μM) or 17β-estradiol (10^-8^M) for 1 h prior to PGE2 (10^-6^M) treatment for another 48 h in hydroxyurea-containing DMEM, and subsequently observed the ability of migration in LoVo cells by migration assay. At the same time, LoVo cells were treated 17β-estradiol (10^-8^M) in the presence or absence of ERs inhibitor, ICI 182780 (5 × 10^-7^M). The responses to different treatments were observed and analysis with a light microscope. *, *p *< 0.05 *versus *control;**, *p *< 0.01 *versus *control. ##, *p *< 0.01 *versus *PGE2 treatment group (mean ± SE, n = 4).

In the present study, we examined the effects of PGE2 on the migration ability in human LoVo colon cancer cells by culturing LoVo cells with PGE2 (10^-6^M) in the presence or absence of JNK1/2 inhibitor (SP600125) for 48 h. Subsequently, we observed the ability of motility in LoVo cells by migration assay. Because DNA synthesis was completely inhibited by hydroxyurea (2 mM), a ribonucleotide reductase inhibitor, present in the medium, the elevated level in cell migration could not be ascribed to the increased potential of cell proliferation. In migration assay (Figure 5), we observed that PGE2 induced a significant increase in cellar migration in LoVo cells. A significant increase of cell migration about 83.78% following PGE2 treatment (10^-6^M) for 48 h was observed in human LoVo cancer cells. However, SP600125 significantly blocked PGE2-induced cell migration about 70.27% when compared with PGE2-treated group. In addition, pretreatment of 17β-estradiol (10^-8^M) significantly inhibited PGE2-promoted LoVo cancer cell migration. ICI 182780 treatment further confirmed the inhibitory property of 17β-estradiol/estrogen receptor (ER) complex on LoVo cellular motility by suppressing function of ERs. These findings suggested that 17β-estradiol might inhibit PGE2-promoted cellular motility by suppressing activation of JNK1/2 in human LoVo colon cancer cells.

## Discussion

**Figure 6:**
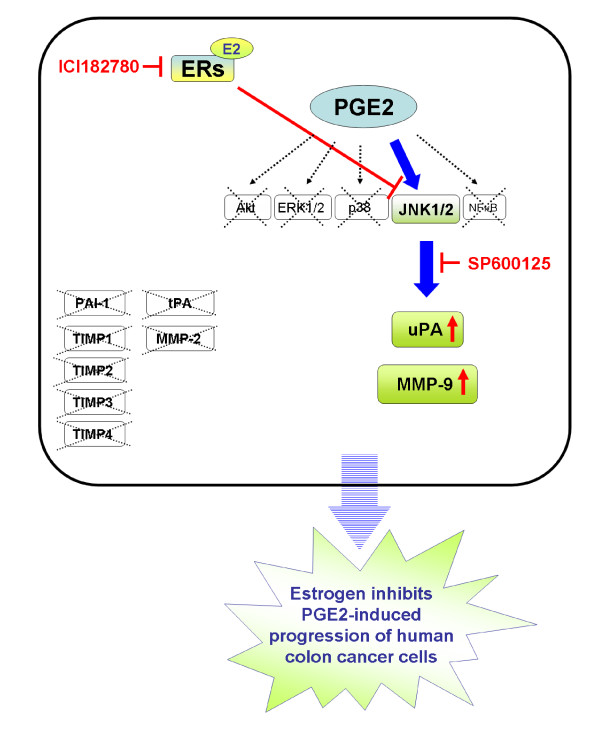
**A schematic representation showing 17β-estradiol inhibition of uPA and MMP-9 expression, and cell motility via suppression of JNK1/2 pathway in human LoVo colon cancer cells**. Administration of prostaglandin E2 (PGE2) rapidly activates kinase such as JNK1/2, thus leading to the expression of downstream targets including uPA and MMP-9, which further promotes cellular motility in human LoVo cancer cells. However, PGE2 treatment shows no effects on regulation of tPA, MMP-2, PAI-1 and TIMPs (TIMP-1, TIMP-2, TIMP-3 and TIMP-4). Estrogen receptor is activated by 17β-estradiol binding to form 17β-estradiol-ER complex. 17β-Estradiol-ER complex presents the properties of anti-cancer by downregulating expression of uPA amd MMP-9 via deactivation of JNK1/2 in LoVo cells. It suggests that 17β-estradiol might inhibit PGE2-induced motility of human LoVo colon cancer cells.

The major findings of the present study can be summarized as followings: **(1) **PGE2 treatment significantly induced phosphorylation of JNK1/2 in human LoVo colon cancer cells. **(2) **Migration of LoVo colon cancer cells was significantly promoted by PGE2 (10^-6^M) treatment. We simultaneously observed that an increase in cell migration was accompanied with the upregulation of migration-related factors including uPA and MMP-9, following PGE2 (10^-6^M) treatment. In addition, JNK1/2 mediated PGE2-induced expression of uPA and MMP-9 in LoVo cells. **(3) **However, PGE2 (10^-6^M) treatment showed no influences on regulating the expression of PAI-1, TIMP-1, TIMP-2, TIMP-3 and TIMP-4 in LoVo cells. **(4) **PGE2-induced expression of uPA and MMP-9 in human LoVo cells was significantly inhibited by 17β-estradiol (10^-8^M) pretreatment. 17β-Estradiol significantly inhibited PGE2-induced uPA and MMP-9 expression by suppressing activation of JNK1/2. These results demonstrate that 17β-estradiol may efficiently inhibit PGE2-induced motility in human LoVo colon cancer cells (Figure 6).

Upregulation of MMPs is reported to contribute to ECM remodeling, tumor cell invasion and metastasis, thus leading to the development of malignant tumor [5]. Both mRNA levels MMP-2 and MMP-9 have been found to be overexpressed in colon carcinomas [21,22]. In the observations of Collins et al. [23], MMP-2 mRNA is more significantly expressed in tumor lesions than in normal colon tissues. Immunostaining assay showed that MMP-9 expression is more frequently present in advanced tumor stages, and in invasive tumor regions wherein cancer cells are in close proximity of inflammatory cells, suggesting that locally proteolytic and collagenlytic activities contributes to the property of invasion in colorectal cancers [24]. In the stage of cancer development, upregulation of MMP-2 and MMP-9 accelerates cell migration and invasion in colorectal cancer [9], thus resulting in the development of malignant tumor, poor prognosis, and shortening disease-free period and overall survival. Here, we founded that administration of 17β-estradiol significantly inhibited PGE2-induced cell migration, and downregulated PGE2-upregulated expression of MMP-9 in LoVo cells. The findings suggested that 17β-estradiol may impair PGE2-promoted cell motility by inhibiting the expression of MMP-9.

Another plasminogen activator system (PAS) with upregulation of uPA and tPA is reported to be involved in MMPs activation and colon cancer development [11,25]. uPA was significantly greater in tumour tissues than normal tissues [26]. uPA upregulated in colon tumor tissue enhances colorectal cancer invasion and metastasis, and this upregulation in uPA is correlated with Dukes's staging and lymphatic invasion [27]. Upregulation in uPA and tPA expression is considered as a marker malignant colon cancer [11,12]. In the present study, we observed that the significant reduction in protein level of uPA was observed after 17β-estradiol treatment in human colon cancer cells. These findings suggested that downregulation of uPA is involved in 17β-estradiol-mediated anticancer effects.

Activation of MMPs is regulated by physiological inhibitors TIMPs [28]. TIMPs not only directly inhibit MMPs but also form complexes with MMPs to control activation and stability of MMPs [10,29]. Four different TIMP species have been identified as TIMP-1, TIMP-2, TIMP-3 and TIMP-4. TIMP binds to MMP in a 1:1 stoichiometric ratio. Induction of functional TIMP-3 in *TIMP-3*-deficient human DLD-1 colon cells shows a growth arrest and inhibits tumor growth *in vivo *[30]. Injection of AdTIMP-2 into preestablished tumors presents the significant reduced tumor growth rates by approximately 60-80% and tumor-associated angiogenesis index by approximately 25-75%. Metastasis of LLC tumor was inhibited by > 90%. In addition, AdTIMP-2-treated mice showed a significantly prolonged survival [31], which emphasizes the importance of endogenous regulation of MMPs activity by TIMPs. In the present study, we further detected the protein level of TIMPs in human LoVo cancer cells that had been exposed to PGE2, which demonstrated that PGE2 treatment shows no influence on regulating TIMP-1, TIMP-2, TIMP-3 and TIMP-4. The results suggested that PGE2 couldn't promote the motility of colon cancer by down-regulating expressions of TIMPs. In addition, the proteolytic action of uPA and tPA is controlled by plasminogen activator inhibitors 1 and 2 (PAI-1 and PAI-2) [11]. We thus simultaneously examined the expression of PAI-1 in LoVo cells that had been treated with PGE2. However, no influence on PAI-1 expression was observed after PGE2 stimulation.

A large number of studies have been dedicated to exploring the molecular mechanisms involved in the downregulation of cancer development. Mitogen-activated protein kinases (MAPKs) include tree major subfamilies such as the extracellularly responsive kinases (ERKs), the c-Jun N-terminal kinases (JNKs), also known as stress-activated protein kinases (SAPKs), and the p38 MAPKs [32]. Studies have showed that ERK1/2 is involved in hepatoma-derived growth factor-induced promotion of carcinogenesis of gastric epithelial cells [33] and in the proliferation of pancreatic stellate cells [34]. p38 MAPK mediates TNFα-induced MMP-9 expression, thus leading to the progression of human urinary bladder cancer cells [35]. JNK/AP-1 signaling pathway may contribute to cellular migration and invasion of prostate cancer cells [36]. Hepatocyte growth factor-activated both Akt and JNK enhance the proteolysis and invasiveness of human nasopharyngeal cancer cells [37]. Abnormal response of NFkB signaling pathway may contribute to the chemoresistance in acute lymphoblastic leukaemia [38]. In the present study, we observed that JNK1/2 signaling pathway mediated expression of uPA and MMP-9 in response to PGE2, which further contributed to cellular motility of human LoVo cancer cells. Previous studies have shown that 17β-estradiol (E_2_) binding to estrogen receptors (ERs) can regulate tissue/cellular responses through multiple signaling pathways [32]. In the present study, we further observed that 17β-estradiol pretreatment inhibited PGE2-induced expression of uPA, MMP-9, and cellular motility via suppressing activation of JNK1/2 in LoVo cells. It suggested that 17β-estradiol presents the anti-cancer properties by inhibiting PGE2-promoted motility in human LoVo cancer cells.

Estrogen receptor α and β (ERα and β) have been identified in colon tissue [15,39,40]. In the normal colon mucosa, there are no significant differences in the protein expression of ERα and β between men and women [40]. Epidemiological data on taking home replacement therapy (HRT) suggests that the loss of estrogen inactivation may be an important mechanism in the pathogenesis of colonic cancer [41]. Studies suggest that estrogen exerts a protective role against the development of fatal colon cancer with a substantially decreased risk in women receiving HRT [16-18], and a reduced mortality from this disease [19]. In this study, we observed that 17β-estradiol reduces PGE2-induced motility of LoVo colon cancer cells derived from male origin. The above study findings strongly suggest that estrogen treatment is an important program against the development of colon cancer.

Estrogen has been reported to modulate the activity of the multiple signaling cascades [42,43] such as the induction of translocation of ER to the cell membrane in MCF-7 cells [44], and then E_2_/ERs complex interact with caveolin-1/-2 in caveolae regions of the plasma membrane [45] wherein cavelin-1 is associated with signaling molecules, such as G proteins, growth factor receptors (IGFIR, EGFR), non-growth factor tyrosine kinase (Src, Ras), and linker proteins (MNAR, striatin) [46,47]. In addition, membrane ER can activate Src-MMP2/9-EGFR-MAPK pathway through Gαi protein in breast cancer cells [47], and activate MAPK-dependent endothelial nitric oxide synthase (eNOS) to increase NO production, contributing to the beneficial effects on cardiac cells [48,49]. In COS7 cell, cytosolic ER binds to membrane IGF-IR and rapidly activates IGF-IR-Ras-Raf-MAPKK-ERK1/2 signaling cascade, which in turn activates ER in a positive feedback loop [50]. In the present study, we observed that 17β-estradiol (10^-8 ^M) treatment rapidly reduces the phosphorylation of JNK1/2 within 15 min. in human LoVo colon cancer cells, which suggests that estrogen-membrane ER complex might majorly reduce PGE2-induced JNK1/2 phosphorylation through non-genomic effect. Previous studies have reported that ERβ is expressed greater in normal colon tissues than pathologic tissues [51], and that the increased ratio of ERα and β due to altered expression of ER subtypes is found in patients with CRC [40], supporting the role of ERβ as a relevant prognostic biomarker of tumor progression [52]. According to these previous studies, we speculated that 17β-estradiol might decrease PGE2-induced cellular motility through ERβ in human LoVo colon cancer cells.

## Conclusions

In summary, we found that PGE2 rapidly activates JNK1/2 kinase, and then increases the protein levels of uPA and MMP-9, which further promotes cellular motility in human LoVo cancer cells. 17β-Estradiol presents the properties of anti-cancer by downregulating expression of uPA and MMP-9 via deactivation of JNK1/2 in LoVo cells. These results also demonstrate that 17β-estradiol efficiently inhibit PGE2-induced LoVo cell motility. These findings might explain that why the incidence and mortality rates of colorectal cancer in women are lower than in men; and that estrogen exerts a protective role against the development of fatal colon cancer with a substantially decreased risk in women receiving hormone replacement therapy (HRT) and a reduced mortality from this disease.

## Abbreviations

E2: 17β-Estradiol; PGE2: Prostaglandin E2; ERK: extracellular signal regulated kinase; p38 MAPK: p38 mitogen-activated protein kinase; JNK: c-Jun N-terminal kinase; NF-κB: nuclear factor κ B; PI3-K: phosphatidylinositol 3-kinase; PKB: protein kinase B; uPA: urokinase plasminogen activator; tPA: tissue plasminogen activator; PAI-1: plasminogen activator inhibitor-1; MMP: matrix metallopeptidase; TIMP: tissue inhibitor of metalloproteinases; DMEM: Dulbecco's modified Eagle's medium; QNZ: 6-amino-4-(4-phenoxyphenylethylamino) quinazoline; GAPDH: glyceraldehyde-3- phosphate dehydrogenase; PBS: phosphate-buffered saline; ECM: extracellular matrix.

## Competing interests

The authors declare that they have no competing interests.

## Authors' contributions

HHH and WSH performed cell culture with drug dose test. YML and WWK performed Immunoblotting assay. LMC and WKC performed cell motility assay. JMH and FJT performed integrity of the data and the accuracy of the data analysis. CJL performed study concept and design, and wrote the manuscript. CYH performed study supervision. All authors read and approved the final manuscript.
